# BoatNet: automated small boat composition detection using deep learning on satellite imagery

**DOI:** 10.14324/111.444/ucloe.000058

**Published:** 2023-05-24

**Authors:** Guo Jialeng, Santiago Suárez de la Fuente, Tristan Smith

**Affiliations:** 1 UCL Energy Institute, The Bartlett School of Environment, Energy and Resources, University College London, London, UK

**Keywords:** object detection, deep learning, machine learning, transfer learning, small boat activity, climate change

## Abstract

Tracking and measuring national carbon footprints is key to achieving the ambitious goals set by the Paris Agreement on carbon emissions. According to statistics, more than 10% of global transportation carbon emissions result from shipping. However, accurate tracking of the emissions of the small boat segment is not well established. Past research looked into the role played by small boat fleets in terms of greenhouse gases, but this has relied either on high-level technological and operational assumptions or the installation of global navigation satellite system sensors to understand how this vessel class behaves. This research is undertaken mainly in relation to fishing and recreational boats. With the advent of open-access satellite imagery and its ever-increasing resolution, it can support innovative methodologies that could eventually lead to the quantification of greenhouse gas emissions. Our work used deep learning algorithms to detect small boats in three cities in the Gulf of California in Mexico. The work produced a methodology named BoatNet that can detect, measure and classify small boats with leisure boats and fishing boats even under low-resolution and blurry satellite images, achieving an accuracy of 93.9% with a precision of 74.0%. Future work should focus on attributing a boat activity to fuel consumption and operational profile to estimate small boat greenhouse gas emissions in any given region.

## Introduction

### Energy crisis, energy security and climate change

The Intergovernmental Panel on Climate Change (IPCC) explains, in its latest report, that humans and nature are being pushed beyond their abilities to adapt due to the anthropogenic emissions caused by economic, industrial and societal activities [1]. Carbon-intensive resources still comprise a large proportion of the energy system [1] – about 80% in 2017 [2]. However, the share of electricity production from renewables increased from 20.8% to 29.0% between 1985 and 2020 [3]. Still, carbon emissions have not been reduced in line with the ambitions of the Paris Agreement, and it is predicted that in the next few years, the gains in carbon reduction due to the Covid-19 pandemic will be erased, faster than expected [4]. However, even under all these pressures and projections, it is still possible for humanity to keep the global temperature below 1.5°C from pre-industrial levels by 2100 if substantial changes are made to the current energy systems.

However, energy security is an important part of the strategies proposed by countries to support economic growth and provide essential services to their populations. Currently, nations deposit most of their energy security into fossil fuels while expanding their renewable power capacity. Fossil fuels and their conversion systems (e.g., internal combustion engines) permit operators to react quickly to changes in the energy demand (i.e., more control over energy deployment) while offering acceptable volumetric energy densities. However, heavy reliance on fossil fuels, coupled with the fuel’s geographical origin, is at the mercy of important price fluctuations due to geopolitical and logistical events, such as Russia’s invasion of Ukraine. These can disrupt global energy systems and affect the stability of nations and human livelihoods [5,6]. On the other hand, renewable energy production and distribution tend to lie within a country’s boundaries. Over the last few years, the price of renewable energy has been catching up with those of subsidised fossil fuels – with some specific examples already undercutting fossil fuel prices [7]. In fact, from 1987 to 2015, the cost of oil and coal rose by approximately 36% and 81%, respectively, and from 1989 to 2015, the cost of natural gas rose by approximately 53% [8]. More recently in March 2022, the UK experienced increases in natural gas to around £5.40/therm, a rise above 1,100% from the price levels seen in 2021 [9]. Nevertheless, it is important to note that renewable energy variability and investment requirements are significant challenges to grid stability and energy security.

### Shipping sector, small boat fleet and emission inventory

Shipping, the backbone of market globalisation, plays an important role in the carbon reduction of human activities as it moves around 90% of all goods around the globe [10]. However, its reliance on fossil fuels, coupled with robust economic growth, saw total carbon dioxide (CO_2_) emissions grow from 962 megatonne (Mt) in 2012 to 1056 Mt in 2018, representing more than 10% of the total global transportation emissions [11]. Furthermore, if nothing is done in the sector, it is projected that by 2050 shipping CO_2_ emissions could grow to 1500 Mt. In this light, the International Maritime Organisation (IMO) produced its ambitions to decarbonise international shipping [12] in 2018. However, this vision only covers international navigation composed of large vessels and does not consider the small boat fleet – vessels below 100 gross tonnages that tend to measure less than 24 m in length [13].

There are good reasons for this decision. First, the IMO focuses mainly on ships that navigate international waters or large ships performing domestic voyages [14]. These vessels are required to have the automatic identification system (AIS) transponders for safe navigation. On the other hand, small boats tend not to have an AIS or a global positioning system (GPS) transponder [15,16], which makes the study of their movements more challenging. Second, small boats are typically registered and monitored by national and regional bodies, and the comprehensiveness of data depends on capital and human resources in addition to the infrastructure to maintain the registry [17]. Third, small boats are a diverse segment of shipping and usage depends on the geographical location, type of activity, construction and operating costs and accessibility to fuel or bunkering infrastructure [18]. Similarly, engine providers are extensive, giving a broad range of fuel consumption curves and emissions [19–21].

Furthermore, fuel selection is equally diverse: petrol, diesel, petrol mixed with engine oil – mainly for two-stroke engines, ethanol and bio-fuels – or a mix of bio-fuel with different fossil fuels. Finally, not all small boats are powered by an internal combustion engine. They can instead be powered by sail, battery-electric or paddles [22–24].

Nevertheless, with all these challenges, the small boat fleet can significantly contribute to the shipping segment’s emission footprint based on its activity [25,26]. Emissions inventories aid our understanding of what measures must be taken to enable governments and industry to start the road to full decarbonisation in a just and equitable way [27–29]. Furthermore, creating effective policies and regulations based on accurate emissions accounting can incentivise the use of energy-efficient technologies, electrification and scalable zero-emission fuels [30,31]. Additionally, if countries want to meet their ambitious decarbonisation emissions targets, they cannot afford to ignore the role played in greenhouse gas (GHG) emissions by the small boat fleet [32–35].

Although it is possible to estimate emissions from large vessels using AIS data sent from a ship’s transponder to be coupled with technical models [11], small vessels depend on the national registration system. Their operation is typically assumed or captured by national fuel sales, which tend to be highly aggregated (e.g., [36]). Developed economies, such as the UK, tend to have a national registry of smaller vessels [37] that provides a sense of their activity level and hence can infer CO_2_ emissions.

However, in developing countries, it tends to be a mixed bag in terms of the level of precision and availability. For instance, in Mexico, only fishing vessels are counted in the national registry [38]. Still, it is not easy to know where they are located and infer their activities. Overall, Mexico does not have a regional CO_2_ inventory specialised in the small boat fleet; instead, they are aggregated as part of the *maritime and fluvial navigation [1A3d]* class in the national annual emission inventory developed by the Instituto Nacional de Ecologá y Cambio Climático (INECC) [39] in a top-down approach based on the IPCC Guidelines [40]. Therefore, quantifying and categorising the small boat fleet will allow a better precision of where and how the emissions are being emitted and will enhance the maritime emission inventories.

Observing shipping activity in the Gulf of California is essential due to its unique geographical location, conformation and biophysical environment [41–43]. Furthermore, the Gulf of California, includes the largest fishing state (Sonora) in Mexico [44] and the most prominent sports fishing destination (Los Cabos, Baja) [45]. Additionally, the region is one of the most protected areas in Mexico due to its diversity of flora and fauna; the area includes the upper part of the Gulf of California, Bahia Loreto and Bahia de los Angeles [46,47].

### Bringing deep learning to small ship detection in satellite imagery

Bringing deep learning, especially convolutional neural networks (CNNs), to the field of satellite image recognition is essential. Satellite image recognition is an important technology for various fields, such as environmental monitoring, natural resource management and disaster response [48–50]. It involves analysing satellite imagery to extract useful information, such as identifying objects, patterns and changes in the earth’s surface. Traditional methods for satellite image recognition rely on hand-crafted features and rules, which can be time-consuming and error-prone [51–53].

Deep learning is a type of artificial intelligence (AI) that has shown great promise in solving complex problems in fields such as computer vision and natural language processing. It involves training large neural networks on vast amounts of data, which allows them to automatically learn complex patterns and relationships [54]. CNNs are a type of deep learning model that is particularly well-suited for image recognition tasks. They can learn hierarchical representations of visual data and can handle large amounts of data, making them efficient and effective for satellite image recognition [55,56].

Recent advances in satellite image recognition using deep learning have shown promising results. For example, researchers have used CNNs to detect objects or patterns in satellite imagery with high accuracy, such as roads, buildings and vegetation [57,58]. They have also applied deep learning to tasks such as land use classification, land cover mapping and disaster damage assessment [59–61].

In conclusion, bringing deep learning, especially CNNs, to the field of satellite image recognition is a large area of opportunity. It allows leveraging the power of AI to automatically learn complex patterns and relationships in satellite imagery. This can lead to improved accuracy, efficiency, automation and scalability compared to traditional methods, and has the potential to benefit a range of fields that rely on satellite imagery data.

### Contributions

The contributions of this study are summarised as follows:

- A purpose-built methodology for this work, BoatNet, was developed. This work shows that BoatNet detects many small boats in low-resolution, blurry satellite images with considerable noise levels. As a result, the precision of training can be up to 93.9%, and detecting small boats in the Gulf of California can be up to 74.0%.
- This work demonstrated that BoatNet could detect the length of small boats with a precision up to 99.0%.
- BoatNet has allowed for a better understanding of the small boat activity and physical characteristics. Based on this, it has been possible to answer questions about the composition of small boats in the Gulf of California. Regarding the authors’ knowledge of the literature, this is a first but essential step in constructing a way, based in object recognition, to estimate the maritime carbon footprint of the small boat fleet.

## Related work

### Small boat fleet and carbon emissions

Previous work related to estimating small-scale vessels without machine learning methods includes using top-down and bottom-up approaches and the use of statistical assumptions.

Parker et al. [62] used a top-down approach to estimate fishing sector emissions in 2011, which reached about 179 Mt carbon dioxide equivalent (CO_2_e), representing 17.1% of the total large fishing ship emissions in that year [63]. However, their work only distinguished between motorised and non-motorised fishing vessels. Greer et al. [64] took a bottom-up approach to classify the fishing fleet in six different sizes, three below 24 m long. The findings show that the small fishing boat fleet in 2016 emitted 47 Mt CO_2_, about 22.7% of the total fishing fleet. Ferrer et al. [65] used an activity-based method using GPS, landing and fuel-used data to estimate the fishing activity around the Baja California Peninsula in Mexico. They found that just the small-scale fishing fleet produced 3.4 Mt of CO_2_e in 2014. To put this into context, Mexico’s national inventory for the domestic shipping sector, but not accounting for fishing activity, in 2014 was recorded at just 2.2 Mt CO_2_e, clearly placing into perspective the role of this fleet segment on national inventories [39].

Several authors have proposed using AIS to monitor the carbon emissions of the fleet [66–70]. Johansson et al. [71] proposed a new model Finnish Meteorological Institute - boat emissions and activities simulator (FMI-BEAM) to describe leisure boat fleet emissions in the Baltic Sea region with over 3000 dock locations, the national small boat registry, AIS data and vessel survey results. However, the method cannot cover countries with no national registry for small boats. Besides, small boats are not just leisure boats. Ugé et al. [72] estimated global ship emissions with the help of data from AIS. They used more than three billion daily AIS data records to create an activity database that captured ship size, speed, and meteorological and marine environmental conditions. This method is highly dependent on AIS data; however, these transponders are not normally installed on board small boats to capture their activity.

Zhang et al. [73] included unidentified vessels in the AIS-based vessel emission inventory. In doing so they developed an AIS-instrumented emissions inventory, including both identified and unidentified vessels. In particular, missing vessel parameters for unidentified vessels were estimated from a classification regression of similar vessel types and sizes in the AIS database. However, the authors did not discuss whether the regression model applies to vessels in most coastal areas. Nor did they explore regional vessel diversity in the database, so statistical inferences and levels of uncertainty about the applicability of their method to other unidentified vessels in a defined single region (e.g., small boats in the Gulf of California, Mexico) cannot be made.

### Convolutional neural network architecture

Neural networks originate from the human perception of the brain. In 1943, American neuroscientists McCulloch and Pitts proposed a theory that every neuron is a multiple-input single-output structure [74]. Furthermore, there are only two possibilities for this output signal: zero or one, which is very similar to a computer.

**Figure 1:**
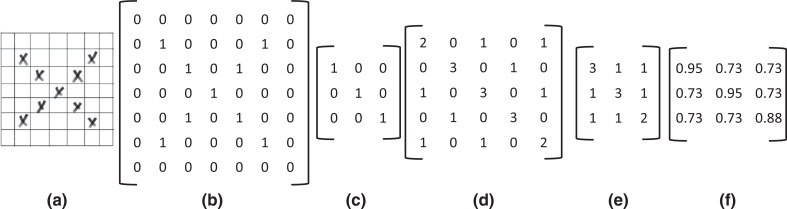
From left to right: (a) Letter X in a 7 × 7 image; (b) letter X in a 7 × 7 matrix; (c) a 3 × 3 convolution kernel; (d) a 5 × 5 feature map; (e) a 3 × 3 feature map after pooling; (f) a 3 × 3 feature map after activating with sigmoid function.

In image recognition, a 7 × 7 image, for example, has 49 elements or cells. If ‘X’ is inputted to the grid, as shown in Fig. 1a, the computer will interpret it as a series of numbers (e.g., zeros and ones) as seen in Fig. 1b. If each cell is either black or white, for example, black can be assigned as one while white would be zero, resulting in a 7 × 7 matrix filled with zeros and ones. After feeding the algorithm as much data as is available, it will be trained to find parameters to determine if the object is an ‘X’ or not. For example, if it is a grey-scale picture, each number is neither zero nor one, but rather a grey-scale value from 0 to 255. If it is a colour image, it will use the red–green–blue (RGB) colour range. Essentially, no matter what the image is, it can be interpreted as a combination number inside a matrix, this eventually working as the input of the neural network. The goal of training a neural network is to find the parameters that make the loss function – it measures how far an estimated value is from its true value – smallest. However, the method described above is time-consuming and computationally expensive to train real-world images. Besides, the algorithm will be hard to recognise once the image is dilated, rotated or changed.

Based on the Neocognitron Model of Fukushima and Miyake [75], LeCun and Bengio [76] invented a practical method for image recognition, called the convolutional neural network. The role of convolution is to use a mathematical method to extract critical features from the image. This is achieved by extracting the features to use a convolution kernel to carry out the convolution operation. The convolution kernel is a matrix, usually 3 × 3 or 5 × 5. For instance, if the convolution kernel is 3 × 3, see Fig. 1c, then a convolution operation will be undertaken with the 7 × 7 ‘X’ matrix (Fig. 1b) and the kernel (Fig. 1c). The operation result is also known as a feature map (Fig. 1d) [77].

The feature map reinforces the features of the convolution kernel. The 3 × 3 convolution kernel portrayed in Fig. 1c has only three oblique blocks of pixels that are ones. So if the original 7 × 7 matrix (Fig. 1b) also has diagonal pixel blocks of ones, the number would be extensive when the convolution operation is complete, which means the desired feature has been extracted. The smaller the value of the pixel block in the other positions of the feature map (Fig. 1d), the less it satisfies the feature. In general, different convolution kernels make it possible to achieve different feature maps.

The next step after convolution is pooling. The pooling method can reduce the feature map size and maintain similar features to the feature map before the pooling process. Figure 1e shows the relatively small feature map after pooling the 5 × 5 matrix (Fig. 1d).

The step after pooling is activation. The activation function decides whether the neuron should be activated by computing the weighted sum and further adding the bias. The essence of the activation function is to introduce nonlinear factors to solve problems that a linear model cannot solve [78]. For example, after activating the sigmoid function, each element in the feature map would be between zero and one, as shown in Fig. 1f.

It is worth noting that the initial convolution kernel may be artificially set. Nevertheless, machine learning will go backwards to adjust and find the most suitable convolution kernel based on its data. As an image generally has many features, there will be many corresponding convolution kernels. After many convolutions and poolings, features can be found, including the diagonal lines of the image, the contours and the colour features. This information is taken and fed into the fully connected network for training, and it is finally possible to determine what the image is.

### Convolutional neural networks in image recognition

The above literature review has demonstrated that the past literature on shipping carbon inventories has not focused on small boats. Thus, the topic of activity-based emission inventories for this segment is an important gap in the literature. There is still considerable work to be done to understand how the small boat fleet operates, what fuels are used, and the level of activity. However, with the development and maturation of a range of computer vision techniques such as CNNs, it may be possible to accurately identify small vessels from open satellite imagery and support understanding of this segment of shipping.

One of the computer vision’s most fundamental and challenging problems is target detection. The main goal of target detection is to determine the location of an object in an image based on a large number of predefined classes. Deep learning techniques, which have emerged in recent years, are a powerful method for learning features directly from data and have led to significant breakthroughs in the field of target detection. Furthermore, with the rise of self-driving cars and face detection, the need for fast and accurate object detection is growing.

In 2012, AlexNet, a deep CNN (DCNN) proposed by Krizhevsky et al. [79], achieved record accuracy in image classification at the ImageNet Large-Scale Visual Recognition Challenge (ILSRVC), making CNNs the dominant paradigm for image recognition. Next, Girshick et al. [80] introduced Region-based Convolutional Neural Networks (R-CNN), the first CNN-based object detection method. The R-CNN algorithm represents a two-step approach in which a region proposal is generated first, and then a CNN is used for recognition and classification. Compared to the traditional sliding convolutional window to determine the possible regions of objects, R-CNN uses a selective search to pre-extract some candidate regions that are more likely to object in order to avoid computationally costly classification and object searches, which makes it faster and significantly less computationally expensive [80,81]. Overall, the R-CNN approach is divided into four steps:

1. Generate candidate regions.
2. Extract features using CNN on the candidate regions.
3. Feed the extracted features into a support vector machine (SVM) classifier.
4. Correct the object positions by using a regressor.

However, R-CNN also has drawbacks: the selective search method is slow in generating positive and negative sample candidate regions for the training network, which affects the overall speed of the algorithm; R-CNN needs to perform feature extraction once for each generated candidate region separately; there are a large number of repeated operations which limits the algorithm performance [82].

Since its inception, R-CNN has undergone several developments and iterations: Fast R-CNN, Faster R-CNN and Mask R-CNN [83–85]. The improvement of Fast R-CNN is the design of a pooling layer structure for the region of interest (ROI). The pooling stage effectively solves the R-CNN operation that crops and scales image regions to the same size, speeding up the algorithm. Faster R-CNN replaces the selective search method with the region proposal network (RPN) [84]. The selection and judgment of candidate frames are handed over to the RPN for processing, and candidate regions are subjected to multi-task loss-based classification and localisation processes.

Several CNN-based object detection frameworks have recently emerged that can run faster, have a higher detection accuracy, produce cleaner results and are easier to develop. Compared to the Faster R-CNN model, the You Only Look Once (YOLO) model can better detect smaller objects, that is, traffic lights at a distance [86], which is important when detecting objects in satellite images. Also, the YOLO model has a faster end-to-end run time and detection accuracy than the Faster R-CNN [86]. Mask R-CNN upgrades the ROI pooling layer of the Fast R-CNN to an ROI align layer and adds a branching FCN layer, the mask layer, to the bounding box recognition for semantic mask recognition [85]. Thus, the Mask R-CNN is essentially an instance segmentation algorithm, compared to semantic segmentation.^1^ Instance segmentation is a more fine-grained segmentation of similar objects than semantic segmentation.

However, even traditional CNNs can be very useful for large-scale image recognition. For example, Simonyan and Zisserman [87] researched the effect of convolutional network depth on its accuracy in large-scale image recognition settings. Their research found that even with small (3 × 3) convolution filters, significant accuracy is achieved by pushing the depth from 16 to 19 weight layers.

In this research, the YOLO framework was selected. It uses a multi-scale detection method, which enables it to detect objects at different scales and to adapt to changes in the size and shape of the objects being observed [88]. Besides, YOLO is highly effective in detecting small objects with high accuracy and precision [89]. This makes it an ideal choice for detecting small objects in satellite imagery contexts, such as small boats in coastal waters. Additionally, YOLO is highly scalable, making it suitable for use in large-scale applications [90].

Finally, this study intends to develop the first stages of BoatNet. This image recognition model aims at detecting small boats, especially leisure and fishing boats in any sea area which, in turn and with further development, could significantly reduce uncertainty in the estimation of small boat fleet emission inventories in countries where access to tracking infrastructure, costly satellite databases and labour-intensive methodologies are important barriers.

## Convolutional neural network configurations

### Target areas in the Gulf of California and dataset statistical analysis

The Gulf of California in Mexico was chosen as an area of study. Ideally, to analyse a sufficient amount of satellite image data, the ports of each of the major harbour cities in the Gulf of California would need to be included in the scope of our study. Thus, the first step in this work was to determine if there was enough satellite data for the area. In this study, the Gulf of California was split into a few zones based on the Mexican state limits: (1) Baja California, (2) Sinaloa, (3) Sonora and (4) Baja California Sur. The satellite dataset used in this analysis included 690 high-resolution (4800 pixels × 2908 pixels) images of ships collected from Google Earth, where the imagery sources are Maxar Techonologies and CNES/Airbus. From the imagery dataset, a statistical analysis was performed on how many times, temporally speaking, the satellite database captured the region of interest. As a result of this analysis, it was found that:

- most cities in the Gulf of California do not have enough open-access satellite data in 2018 and 2021, while many cities have relatively rich satellite data between 2019 and 2020;
- there has been a steady increase in the collection of satellite data in the Gulf of California from 2018 to 2020;
- the open-access and high-quality satellite data from Google Earth Pro is not immediately available to the public;
- differences in data accessibility are still evident among different cities. For example, Guaymas in the state of Sonora has rich satellite images in 2019 and 2020. However, other cities, such as La Ventana in the state of Baja California Sur, did not appear on Google Earth Pro between 2019 and 2020.

**Figure 2:**
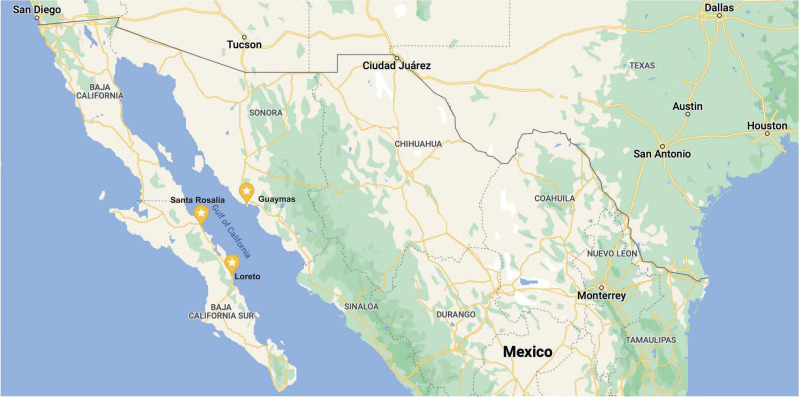
The geographic locations of the three target cities – Santa Rosalia, Loreto, and Guaymas. (Source: Google Maps 2021.)

**Figure 3:**
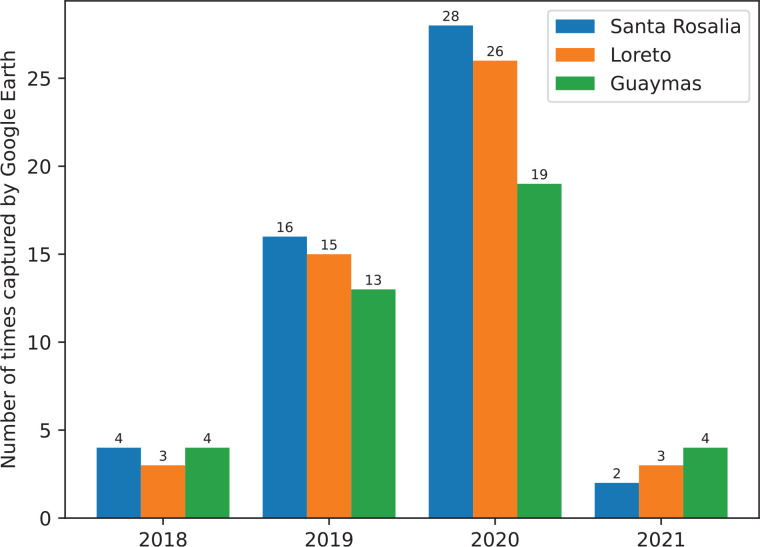
Number of times the three cities (Santa Rosalia, Loreto, Guaymas) were captured by Google Earth Pro from 2018 to 2021.

For this reason, continuing with the previous strategy of analysing the satellite data for each city in the Gulf of California would lead to a relatively large information bias and thus would not achieve an effective object detection model. Therefore, the following three cities with the richest data-accessibility in Google Earth Pro were chosen as the target areas for this study: Santa Rosalia, Loreto and Guaymas (see Fig. 2 for their geographical locations). The number of times captured by Google Earth Pro [91] is shown in Fig. 3 with a database of 583 images with timestamps between 2019 and 2020 for the three Mexican coastal cities.

### Preprocessing

Each satellite image used for training was manually pre-labelled with a highly precise label box [92]. The original dataset contained images larger than 9 MB, which is an efficiency burden for neural network training, especially when few objects are detected. For this reason, all images were resized from 4800 pixels × 2908 pixels to 416 pixels × 416 pixels, with the file sizes reduced to between 10KB and 40KB [93].

Each satellite image for targeting or testing can be directly extracted from Google Earth Pro. Before downloading these images, a few things were done initally. First, all the layers from Google Earth Pro needed to be removed. Then, it was necessary to open the ‘Navigation’ tab of the ‘Preferences’ menu; ‘Do not automatically tilt while zooming’ needs to be clicked. This allowed the images available to be acquired which were directly above sea level. Finally, the eye altitude was set to 200 m and the images were saved in 4800 pixels × 2908 pixels.

### Single object detection architecture

**Figure 4:**
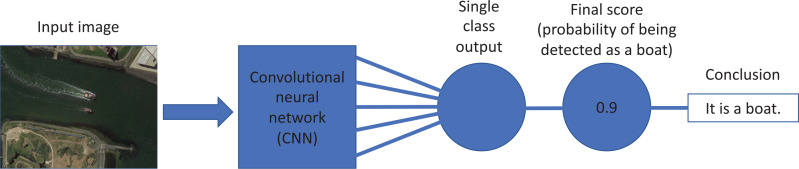
Model architecture. Detection model architecture for obtaining a conclusion from an input satellite image of boats. Images are preprocessed and passed through a CNN. The model’s output is a score, *y* ∈ (0,1), representing the probability of being detected as a boat.

**Figure 5:**
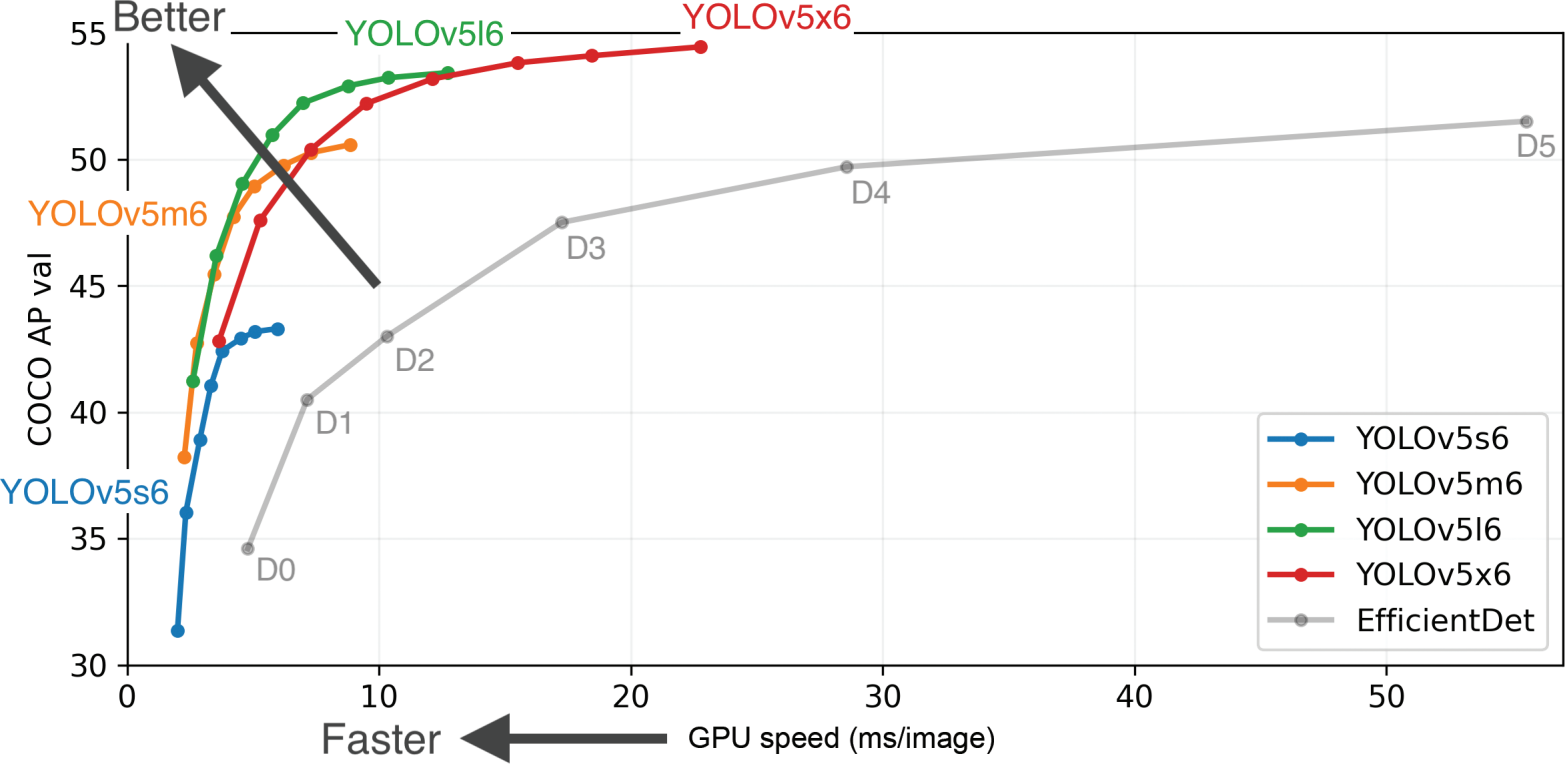
Average Precision (AP) versus GPU Speed in the 6th generation of YOLOv5 model under COCO data set [86,94].

Figure 4 shows a schematic of the models being used for detecting boats, where satellite images in the Gulf of California are the input of a pre-trained CNN. The detection accuracy was determined by computing the mean probability score from the Gulf’s satellite images. In the section Convolutional neural networks in image recognition, recent literature and the development of CNNs, including the YOLO model, were discussed. YOLO version 5 (YOLOv5) has four different categories of models, YOLOv5s, YOLOv5m, YOLOv5l and YOLOv5x [94]. They have 7.3 million, 21.4 million, 47.0 million and 87.7 million parameters, respectively. The performance charts can be seen in Fig. 5, which shows that the YOLOv5l model can achieve higher average precision with the same faster computing speed. Thus, in this study, Google Colab’s Tesla P100 GPU^2^ and the YOLOv5 framework were used.

Satellite images often contain noise such as shadows cast by water on the sea surface or haze clouds in the atmosphere, which make the training data inaccurate and often cause problems ensuring the model’s correctness. He et al. [95] proposed a simple but effective image prior-dark channel before removing haze from a single input image. The prior-dark channel can be used as a statistic of outdoor haze-free images. Based on critical observation, most local patches in outdoor haze-free images contain some pixels whose intensity is very low in at least one colour channel. Using this prior-dark channel before the haze imaging model, the thickness of the haze can be estimated, and a high-quality haze-free image can be recovered. Moreover, a high-quality depth map can also be obtained as a byproduct of haze removal. In the same way, shadows can be removed using the prior-dark channel.

Similar to the principle of using convolution kernels, specific image kernels can sharpen the image. While the sharpening kernel does not produce a higher-resolution image, it emphasises the differences in adjacent pixel values, making the image appear more vivid. Overall, sharpening an image can significantly improve its recognition accuracy with a 5 × 5 image kernel.

### Object measurement and classification

Measuring the length of a ship was one of the most challenging topics in this study. As Google Earth Pro does not provide an application programming interface (API) for accurate scales, manually measuring the size of a particular scale became the core process to calculate the size of any given ship. To achieve that it is important that all of the captured satellite images have the same eye altitude. By measuring only one real length of the object through the Google Earth Pro measurement tool and knowing the pixel length of this object, the length of one pixel in the satellite image of the fixed eye altitude can be calculated.

As the dataset for the training model was created with each edge tangent to the edge of the detected object, it can roughly treat the boat’s length as the length of the diagonal within the detection box. Second, as the scale is central to the detection of the small boat fleet, the imagery scale should adhere to the following rules:

- Cannot be too large. The image should contain the full area in which boats may be found.
- Cannot be too small. If this is not followed it is highly probable that the group of boats are detected as a single but larger boat.
- Be sufficiently clear. This characteristic allows the algorithm to quantify the boat’s length and accurately classify the measurements.

**Figure 6:**
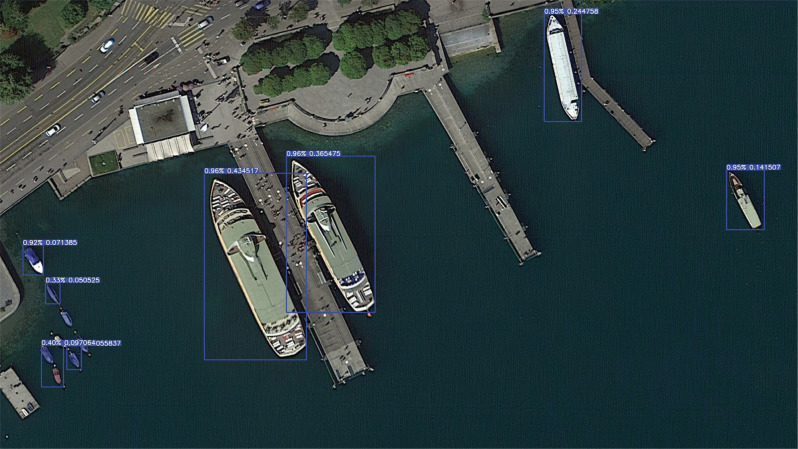
An image from Google Earth Pro for Zurich Lake on 16 August 2018 when eye alt is 200 m. (Source: Google Earth Pro, 2021.)

The eye altitude was set to 200 m based on the above rules. This study used a satellite image of Zurich Lake, Switzerland, on 16 August 2018 as the standard image for defining the scale (Fig. 6). Compared with other regions, the satellite image of Zurich Lake complies with the rules, and it is a suitable candidate as the standard for measuring the length of small boats. This standard was then used for the rest of the imagery database.

A length measurement comparison was made between the BoatNet framework and Google Earth Pro to validate the length method. The vessel in Fig. 6 had a YOLO length of 0.43 that, after the scale conversion, represented 55.17 m. With an eye altitude of 200 m, the ratio of the absolute length to the YOLO length was approximately 127. Finally, after several verification tests, this ratio returned a small margin of error (around 1–3%) and hence was deemed a suitable scaling ratio for the remaining images. Moreover, having the same ratio and eye altitude was not enough. The resolution of each image must be the same, so measurements are standardised. For this purpose, all datasets that detect small boats will maintain a resolution of 3840 × 2160 pixels.

In a certain sense, large vessels (e.g., cargo ships) and small vessels (e.g., small boats for domestic use) are distinguished when creating the dataset for the area of interest. However, due to the scaling, some large vessels such as general cargo ships do not appear fully in an image. Hence they are not considered in the statistical results of this work. On the other hand, some of the larger vessels, slightly shorter in length than the 200 m eagle eye scale, are identified correctly by the algorithm and counted as part of the number of large vessels in the area. A Python script was then designed to count the number of small and large boats between regions.

After distinguishing between large and small boats, it is necessary to distinguish between small recreational boats for domestic use and fishing boats. The model used the detected deck colour of the small boat to distinguish between them. If the deck was predominantly white, it was assigned as a recreational boat, while any other mix of colour would be designated as a fishing boat. It is recognised that this is a broad and simplistic classification method, but it is an effective one to test the categorisation power of the model. Each of the detected boats (i.e., the objects within the four coordinate anchor box) were analysed whether the colour was white or mainly white to assign it to each category. As a final step, the model performed the category counting for each image, where a Python script was designed to count the small white boats.

## Results

### Train custom data: weights, biases logging, local logging

**Figure 7:**
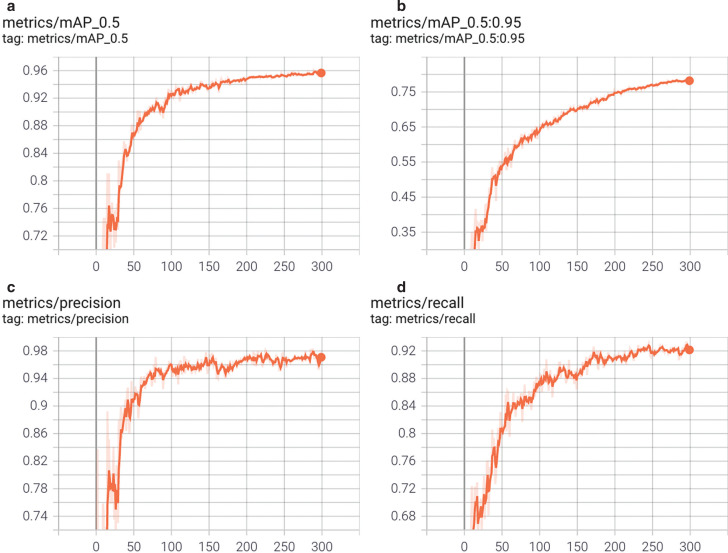
Average model precision when IOU is larger than 0.50; average model precision when IOU is between 0.50 and 0.95; The model precision; The model recall rate.

As shown in Fig. 7, the average accuracy, precision and recall of the model all show a significant increase with the model training number when the intersection over union (IOU)^3^ is between 50% and 95%. In particular, the precision of the model can eventually reach a level close to 96%. However, this does not necessarily mean that the model will also fit the satellite imagery of the Gulf of California. First, such high-accuracy results only tell us that the model can achieve a relatively high recognition accuracy, which gradually increases and reaches 96% after 300 training repetitions. In the case that the algorithm needs to be trained for this area, consideration must be given to purposefully selecting many small boats in or near the area as a data source for training the model.

To train models faster, the images’ resolution was reduced by about 70 times, resulting in images of 416 pixels × 416 pixels. The training could otherwise take two weeks if the images used had a resolution of 4800 pixels × 2908 pixels.

**Figure 8:**
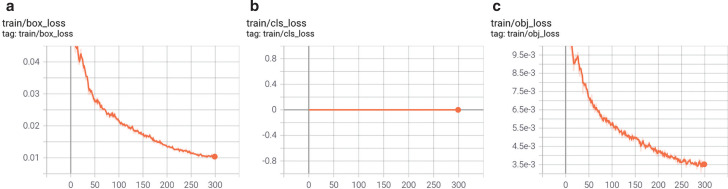
The box loss rate of the model; the class loss rate of the model; the object loss rate of the model.

**Figure 9:**
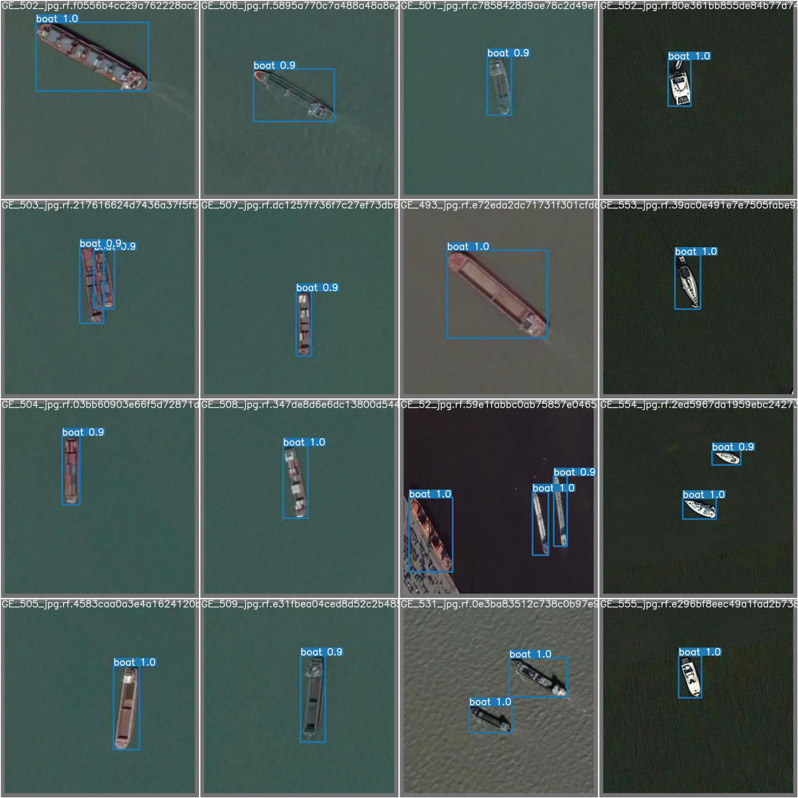
Test result of a trained model for detecting ships. (Source: Author-originated, based on Google Earth Pro imagery, 2021).

Similarly, as shown in Fig. 8, the loss rate of the box can eventually reach 1% as the number of training sessions increases. As this study has defined only one class of object (i.e., boat), the probability that the detection box does not detect that it is a boat at all is 1%. Similarly, because there is only one class, the class loss rate is zero. Figure 9 presents the prediction results during the training of the model, and shows that the model can detect the presence of vessels in 100% of the tested ranges and gives the corresponding range boxes. Most detected boats have a 90% probability of being boats, an acceptable value for object detection. As only one class was set, some were also considered a 100% probability of being boats.

### Detection results and small boat composition

**Figure 10:**
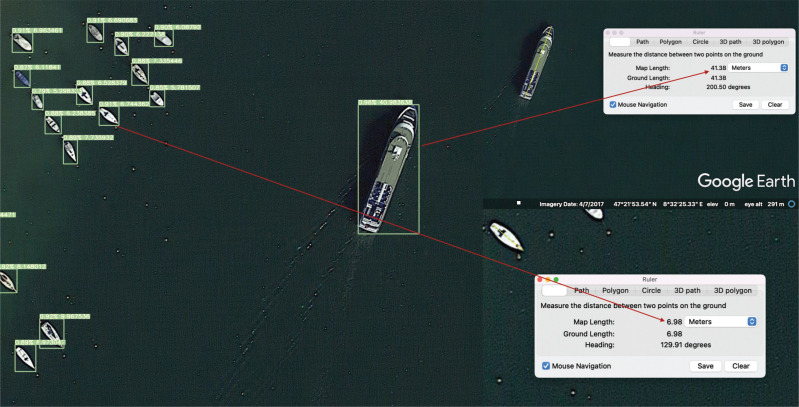
Comparing the length of boats using Google Earth ruler and computer vision algorithm. This example shows the image from Google Earth Pro for Zurich Lake on 16 August 2018 when the eye alt was 200 m. (Source: Author-originated, based on Google Earth Pro imagery, 2021).

Starting with the length measurement comparison of a detected small boat and a large ship by BoatNet against Google Earth Pro measuring tools, Fig. 10 shows that the small boat detected measured 6.98 m using Google Earth Pro, while BoatNet estimated 6.74 m. The error between them is 3.4%. Google Earth Pro measured the larger ship at 41.38 m and BoatNet at 40.98 m. The error between them is less than 1.0%.

**Figure 11:**
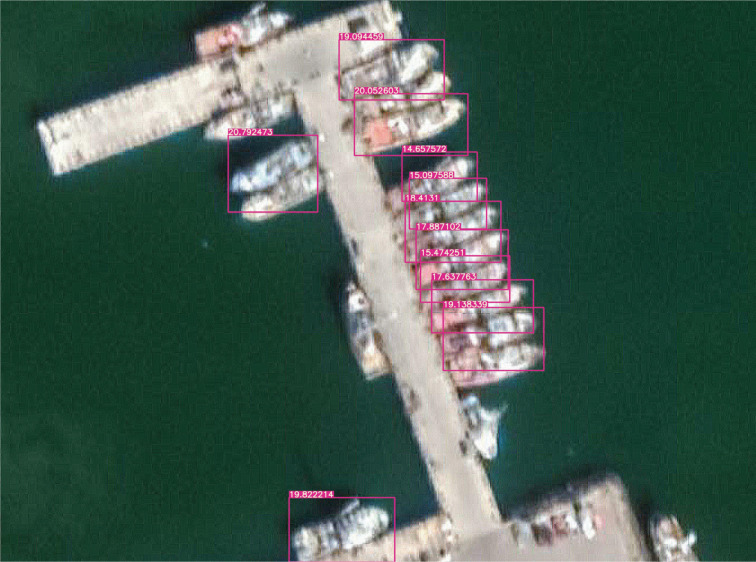
When small boats are moored closely together in the harbour, the model may recognise two small boats as one. The image is from Guaymas, January 2020. (Source: Author-originated, based on Google Earth Pro imagery, 2021).

**Figure 12:**
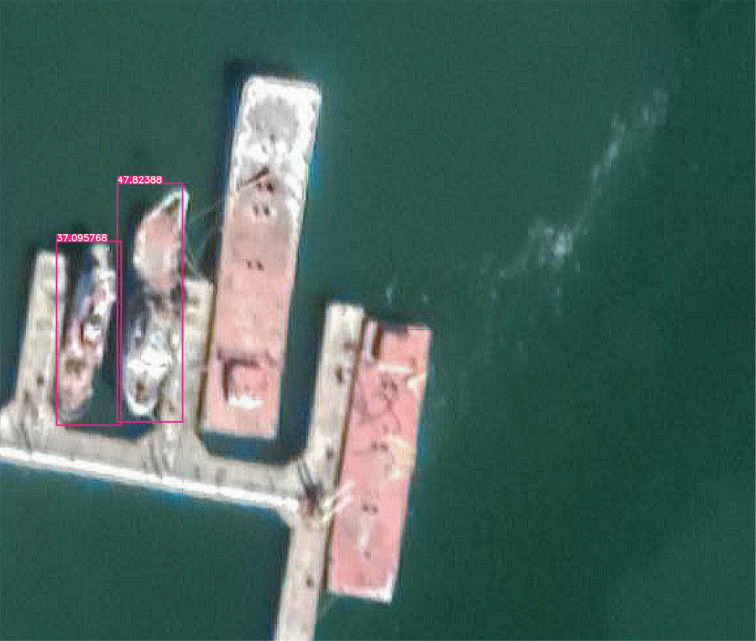
When the cargo ship is full of cargo, the ship looks like a rectangular jetty from above and loses the normal shape of a ship. The image is from Guaymas, January 2020. (Source: Author-originated, based on Google Earth Pro imagery, 2021).

**Figure 13:**
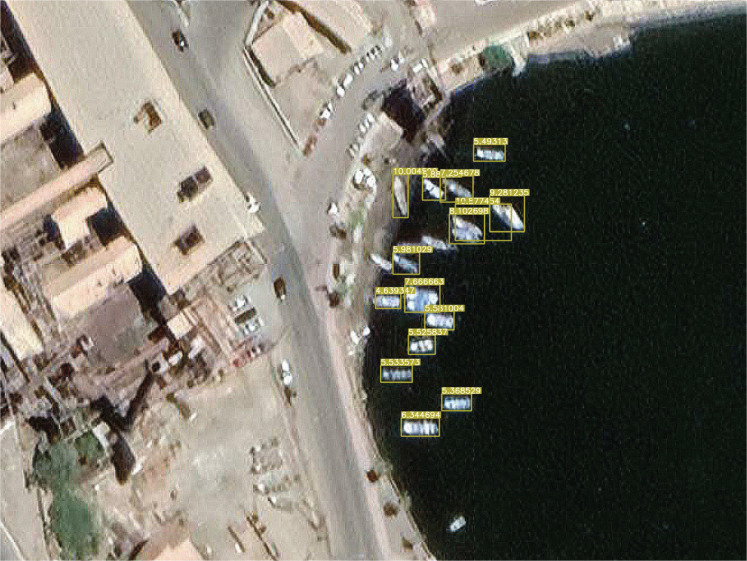
Models may have difficulty detecting small boats moored on the beach. The image is from Santa Rosalia, February 2021. (Source: Author-originated, based on Google Earth Pro imagery, 2021).

As explained in the section Single object dectection architecture, it was unproductive for the model to select the entire region for the study due to the varying amount of publicly available regional images from Google Earth Pro over the past three years. Ultimately, the satellite image database was built from 690 images. However, as stated in that section, some of the slightly earlier satellite images offered inferior detail representation capabilities, which resulted in the model not accurately detecting the target’s features. To improve model accuracy, an image enhancement process using a 5 × 5 sharpening kernel allowed for a higher recognition rate. However, the following situations still occur:

1. Figure 11: When the detailed representation of the image is indigent, that is, the images are blurred, and two or three small boats are moored together, the model is very likely to recognise the boats as a whole. There are two reasons for this problem. First, the training data is primarily a ‘fuzzy’ data source. Thus, when two or three small boats are moored together, the model cannot easily detect the features of each small boat individually. In contrast, it may seem more reasonable to the model that the boats as a whole have the same features. The second reason is that most data sources are individual boats on the surface or boats docked close to each other. As the data sources do not fully consider the fuzzy nature of the detail needed to detect the object and the fact that they are too close together, the model naturally does not recognise such cases.
2. Figure 12: When a large cargo ship is moored, the ship appears as a ‘rectangle’ from the air, much like a long pier, and is sometimes undetectable because small vessels with a rectangular shape were not common at the time the data feed was compiled. This also applies to uncommon vessels such as battleships. This could be corrected if the model considered larger ships, but this was outside the scope of this work.
3. Figure 13: The recognition rate was also significantly lower when the boats sometimes lay on the beach rather than floating on the water. This is because most of the training data are based on images in the water rather than boats on the beach.

**Figure 14:**
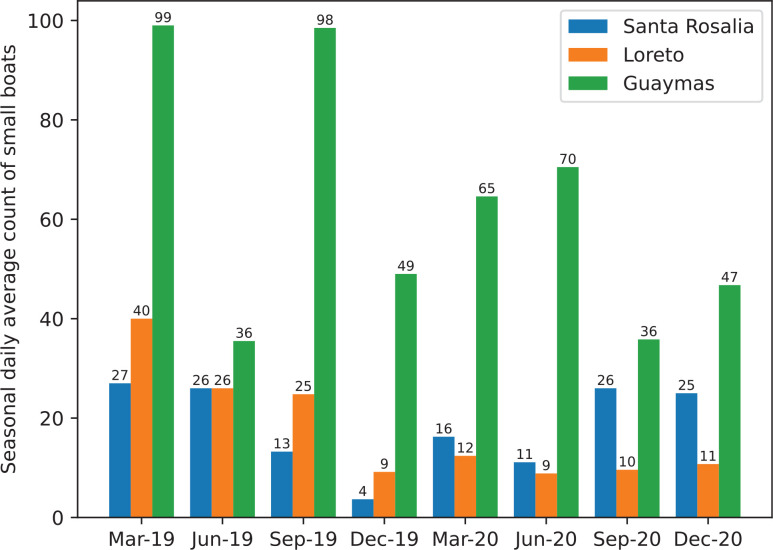
The seasonal daily average count of small boats in Santa Rosalia, Loreto and Guaymas between 2019 and 2020.

**Figure 15:**
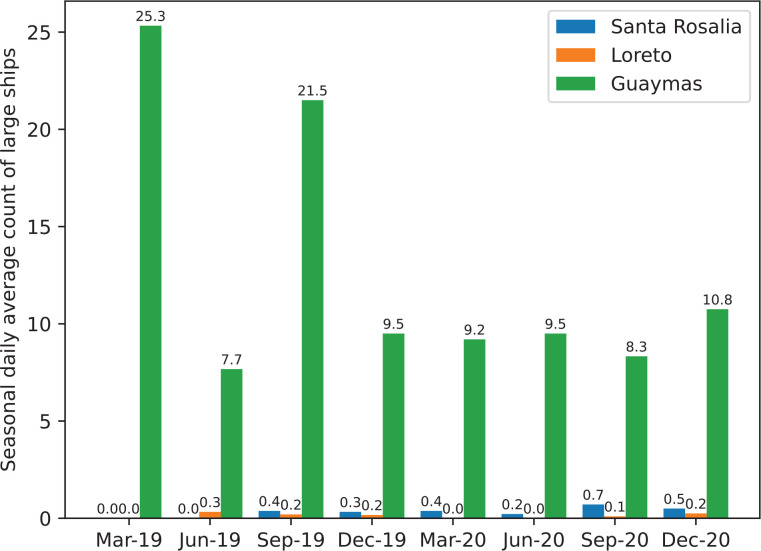
The seasonal daily average count of large ships in Santa Rosalia, Loreto and Guaymas between 2019 and 2020.

Nevertheless, as Figs 11–13 demonstrate, the model still detects most small boats in poorly detailed satellite images, even those that the human eye cannot easily detect. The number of small and large ships between regions can be seen in Figs 14 and 15. Two different types of port cities are exemplified by Guaymas, Loreto and Santa Rosalia:

1. Santa Rosalia and Loreto have a much smaller number of small boats and almost no large ships.
2. The port of Guaymas presented a larger number of small boats when contrasted to the other two coastal cities. There were between 1.37 and 8.00 times more small boats detected in Guaymas than in Santa Rosalia (dependent on the month and year of the image) and 3.00 times more than in Loreto.
3. Guaymas also had a larger number of large ships. There were more than 10 times more large ships detected in Guaymas than in Santa Rosalia and Loreto.
4. In the relatively large seaports of Guaymas and Loreto, there is a tendency for both large and small vessels to decrease with time.

In fact, according to statistics [96] from the Mexican government, in 2020, the populations of Guaymas, Loreto and Santa Rosalia were 156,863, 18,052 and 14,357, respectively. Therefore, the results infer that the number of detected boats is correlated to the number of habitats, which makes sense as, by probability, there would be more economical and leisure activities around larger coastal cities.

### Leisure and fishing boats in the Gulf of California

According to the statement in the section Single object dectection architecture, determining whether a boat is white can be used as a criterion to determine whether a boat is used for recreation or fishing. As seen in Table 1, most of the small boats captured in the photo of Guaymas in 2020 are white (i.e., all can be classified as recreational boats). However, as previously discussed, this conclusion is limited as the algorithm does not consider, for example, other colours as part of the characteristics of leisure boats. Furthermore, although there are many uncertainties in detecting the colour of the boats, the algorithm also considers situations where the colour is not fully white due to atmospheric refraction, weather conditions or cloud interference. The algorithm also considers cases where the boat’s colour is light. Therefore, this approach is acceptable from the point of view of algorithm complexity, results and detecting data quality.

**Table 1.** Detection example. Small boats, large ships and small white boats in Guaymas in 2020

	Mar 20	Jun 20	Sep 20	Dec 20
Small boats	323	283	215	187
Large ships	46	38	50	43
Small white boats	302	273	210	174
Shipping boats	21	10	5	13

## Discussion

This study demonstrated the capabilities of a deep learning approach for the automatic detection and identification of small boats in the waters surrounding three cities in the Gulf of California with a precision of up to 74.0%. This work used CNNs to identify types of small vessels. Specifically, this study presented an image detection model, BoatNet, capable of distinguishing small boats in the Gulf of California with an accuracy of up to 93.9%, which is an encouraging result considering the high variability of the input images.

Even with the model’s level of performance using large and highly ambiguous training images, it was found that image sharpening improved model accuracy. This implies that access to better quality imagery, such as that available through paid-for services, should considerably improve model precision and training times.

The results of this research have several important implications. First, the study used satellite data to predict the number and types of ships in three important cities in the Gulf of California. The resulting analysis can contribute to the region’s shipping fleet composition, level of activity and ultimately their carbon inventory by adding the emissions produced by the small boat fleet. Furthermore, through this approach, it is also possible to assign emissions into regions supporting the development of policies that can mitigate local GHG and air pollution. In addition, the transfer learning algorithm can be pre-trained in advance and immediately applied to any sea area worldwide. This will provide a potential method to increase efficiency for scientists and engineers worldwide who need to estimate local maritime emissions. In addition, the model can quickly and accurately identify the boat’s length and classify them, allowing researchers to allocate more time to the vessels they need concentrate on, not just small boats. Finally, all of the above benefits can be exploited in under served areas with a shortage of infrastructure and resources.

This work is the first step to building emission inventories through image recognition, and it has some limitations. The study considered the ship as a single detection object. It did not evaluate whether the model can improve the accuracy of identifying ships in the case of multiple detection objects. For instance, BoatNet was not trained to detect docks to improve the metrics of detecting boats. By down-sampling the image to 416 pixels × 416 pixels, it is possible to mask some of the boats at the edges of the photograph. Furthermore, deep learning models train faster on small images [97]. A larger input image requires the neural network to learn from four times as many pixels, increasing the architecture’s training time. In this work, a considerable proportion of the images in the dataset were large images of 4800 pixels × 2908 pixels. Thus, BoatNet was set to learn from resized small images measuring 416 pixels × 416 pixels. Due to the low data quality of the selected regions, the images are less suitable as training datasets. However, using datasets from other regions or higher-quality open-source imagery may result in inaccurate coverage of all types of ships in the region. When focusing on the small boat categorisation and the data used, understanding the implications of different environments (e.g., water or land) on object classification accuracy through the AI fairness principle deserves further study. From this point of view, large-scale collection of data sources in the real physical world would be costly and time-consuming. That said, it is possible that reinforcement learning, or building simulations in the virtual world, could reduce the negative impact of the environment on object recognition and thus improve its categorisation precision. Of all these limitations, model detection still achieves excellent performance in detecting and classifying small boats. To enrich the analysis, one of the future works planned is comparing research results with different algorithms for the same problem.

It is important to remember that BoatNet currently only detects and classifies certain types of small boats. Therefore, to estimate fuel consumption and emissions, it is necessary to couple it with small boat behaviour datasets [65], typical machinery, fuel characteristics and emission factors unique to this maritime segment [39].

Finally, this work has demonstrated that deep learning models have the potential to identify small boats in extreme environments at performance levels that provide practical value. With further analysis and small boat data sources, these methods may eventually allow for the rapid assessment of shipping carbon inventories.

## Data Availability

The datasets generated during and/or analysed during the current study are available in the repository: https://github.com/theiresearch/BoatNet.

## Notes

1 In computer vision, image segmentation is the process of partitioning an image into multiple image segments, also known as image regions or image objects.

2 Colab Pro limits RAM to 32 GB while Pro+ limits RAM to 52 GB. Colab Pro and Pro+ limit sessions to 24 hours.

3 IOU is an evaluation metric used to measure the accuracy of an object detector on a particular dataset.
